# Refractive Index Sensor Based on the Fano Resonance in Metal–Insulator–Metal Waveguides Coupled with a Whistle-Shaped Cavity

**DOI:** 10.3390/mi13101592

**Published:** 2022-09-25

**Authors:** Bo Li, Huarong Sun, Huinan Zhang, Yuetang Li, Junbin Zang, Xiyuan Cao, Xupeng Zhu, Xiaolong Zhao, Zhidong Zhang

**Affiliations:** 1 School of Software, North University of China, Taiyuan 030051, China; 2 Key Laboratory of Instrumentation Science & Dynamic Measurement of Ministry of Education, North University of China, Taiyuan 030051, China; 3 School of Physical Science and Technology, Lingnan Normal University, Zhanjiang 524048, China; 4 School of Electrical and Control Engineering, North University of China, Taiyuan 030051, China

**Keywords:** plasmonic, refractive index sensor, finite element method, Fano resonance, metal–insulator–mental

## Abstract

A plasmonic refractive index sensor based on surface plasmon polaritons (SPPs) that consist of metal–insulator–metal (MIM) waveguides and a whistle-shaped cavity is proposed. The transmission properties were simulated numerically by using the finite element method. The Fano resonance phenomenon can be observed in their transmission spectra, which is due to the coupling of SPPs between the transmission along the clockwise and anticlockwise directions. The refractive index-sensing properties based on the Fano resonance were investigated by changing the refractive index of the insulator of the MIM waveguide. Modulation of the structural parameters on the Fano resonance and the optics transmission properties of the coupled structure of two MIM waveguides with a whistle-shaped cavity were designed and evaluated. The results of this study will help in the design of new photonic devices and micro-sensors with high sensitivity, and can serve as a guide for future application of this structure.

## 1. Introduction

Surface plasmon polaritons (SPPs) have the capability of overcoming the diffraction limit due to their energy evanescently confining in the perpendicular direction of the metal–insulator interference [1,2,3]. SPPs have been widely used in surface-enhanced Raman scattering, imaging, solar cells, sensors, optical filters [4,5,6,7,8,9,10], and so on. Recently, another new type of surface plasmon, Tamm plasmon modes in the metal-PhC cavity, has shown excellent performance in gas sensors and refractive index sensors due to its sensitivity to the environment and strong localization [11,12,13]. For the application of manipulating SPPs in chip-scale integration, metal–insulator–metal (MIM) waveguides are an excellent subwavelength photonic device [14,15]. A plasmonic interferometric biosensor based on MIM waveguides for phase-sensitive biomolecular analysis has been proposed [16]. SPP photonic devices based on MIM waveguides have received increasing attention, such as all-optical switches, sensors, and slow light devices [17,18,19].

Recently, the Fano resonance phenomenon was observed in the MIM waveguide-coupled resonator system [20,21]. It was first discovered by Fano Ugo, and has an asymmetric line profile due to the interference between a narrow discrete resonance and a broad spectral line or continuum [22,23,24]. Fano resonance, as a weak coupling and interference phenomenon, has a unique line shape, which provides a promising pathway to achieve ultrahigh sensitivity sensors, lasing, all-optical switching, and nonlinear and slow light [25,26,27,28,29,30,31]. Biosensors and chemical sensors based on Fano resonance have attracted much attention from researchers due to their extreme sensitivity to changes in structural parameters and the surrounding dielectrics [32,33]. These sensors exhibit good performance in terms of sensitivity and figure of merit (FOM) [34,35]. To achieve ultrahigh sensitivity, these structures and parameters need to be optimized. Therefore, the way in which to optimize the MIM waveguide-coupled resonator system to obtain a plasmonic coupled system with a Fano line shape is a key issue for designing high-sensitivity plasmonic sensors.

In this study, a whistle-shaped plasmonic structure composed of one whistle-shaped cavity and an MIM waveguide was designed to obtain high-sensitivity sensors based on Fano resonance. The transmission properties and magnetic field distributions of the whistle-shaped structures were simulated using the finite element method (FEM). The effects of the structural parameters of the whistle-shaped structure on the transmission spectrum were investigated. The refractive index sensitivity and the FOM of the whistle-shaped structure were explored. A derived plasmonic structure composed of a double whistle-shaped coupled structure was designed and evaluated. The sensitivity of the derived structure was examined.

## 2. Structure Model and Analytical Method

**Figure 1:**
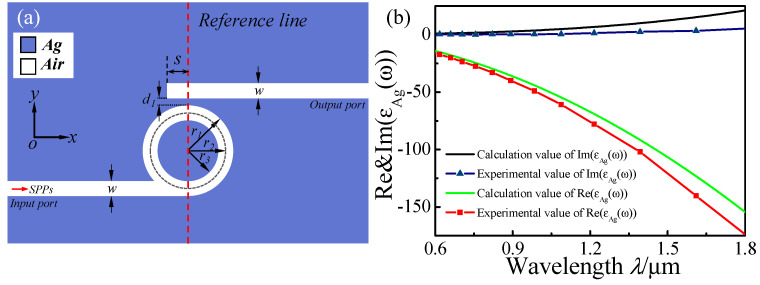
(**a**) Two-dimensional schematic for the MIM waveguides coupled with a whistle-shaped cavity. (**b**) The calculated and experimental values of the real and imaginary parts of the permittivity of silver.

As shown in Figure 1a, the 2D schematic of the proposed plasmonic structure is composed of a whistle-shaped cavity and an MIM waveguide. The whistle-shaped cavity was composed of the input MIM waveguide on-interval and a ring cavity. As shown in Figure 1, the blue and white parts denote Ag (*ε_m_*) and air (*ε_s_*), respectively. The widths *w* of these MIM waveguides were fixed at 50 nm to support the only fundamental transverse magnetic (*TM*_0_) in the MIM waveguides. The length of the output MIM waveguide is *L*, and the gap between the output MIM waveguide and the ring cavity is *d_1_*. The inner and outer radii of the ring cavity in the whistle-shaped structure are represented by *r*_1_ and *r*_3_, and the center radius of the ring cavity is *r*_2_ *= (r*_1_ *+ r*_3_)/2. The red vertical dashed line passes through the center of the ring cavity and is defined as the reference line.

The frequency-dependent complex relative permittivity *ε(ϖ)* of silver is characterized by the modified Debye–Drude dispersion mode [36,37] as follows:

(1) $$\varepsilon (\varpi )=\frac{\varepsilon_{\infty }+(\varepsilon_{s}-\varepsilon_{\infty })}{1+i\varpi \tau }+\frac{\sigma }{i\varpi \varepsilon_{0}}$$

where *ε*_∞_ = 3.8344 is the infinite frequency permittivity, *ε_s_* = −9530.5 represents the static permittivity, *σ* = 1.1486 × 10^7^ S/m is the conductivity, and *τ* = 7.35 × 10^−15^ s is the relaxation time. Figure 1b shows the real part and the image part of the experiment value and modified value using the Debye–Drude model for the silver relative permittivity. The theory mode is in good agreement with the experimental data.

The transmission properties of the single whistle-shaped structure and the double whistle-shaped coupled derived structure were investigated using FEM with perfectly matched layer-absorbing boundary conditions. The transmittance is defined as *T = |S*_21_*|^2^*, where *S*_21_ is the transmittance of the MIM waveguide [38].

For MIM waveguides, only the fundamental transverse magnetic (TM_0_) mode can be supported, and its dispersion relation is expressed as [39]:

tanh(*κw*) = −2*κp*α/(*κ*^2^ + *p*^2^α^2^) (2)

where *κ* and *w* are the perpendicular core wave vector and the insulator width of the MIM waveguide, respectively. The symbols in Equation (1) are defined as *p* = *ε*_in_/*ε*_m_ and *α* = [*k*_0_^2^(*ε*_in_ − *ε*_m_) + *κ*]^1/2^, where *ε*_in_ and *ε*_m_ are the dielectric constants of the insulator and the metal, respectively; *k*_0_ = 2π/*λ*_0_ is the free space wave vector; *κ* can be solved from Equation (1) using the iterative method. Thus, the effective index *n*_eff_ of the MIM waveguide can be defined as *n*_eff_ = (*ε*_m_ + (*κ*/*k*_0_)^2^)^1/2^. The wavelength of SPPs, *λ*_spp_, can be expressed as *λ*_spp_ *= λ*_0_/Re (*n*_eff_), where Re (*n*_eff_) is the real part of *n*_eff_.

## 3. Results and Discussions

**Figure 2:**
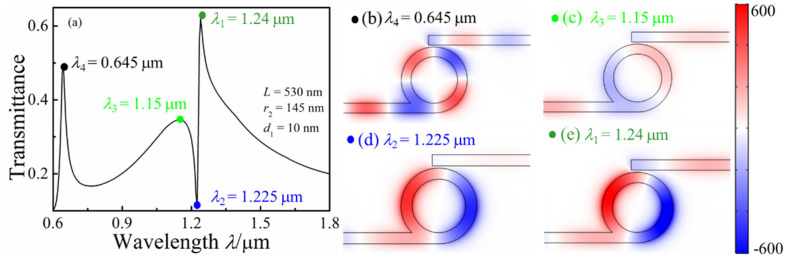
(**a**) Transmission spectra of the MIM waveguide-coupled whistle-shaped cavity; (**b**) contour profiles of the normalized *H*_z_ field distributions of the MIM waveguide whistle-shaped cavity: (**b**) *λ* = 0.645 mm, (**c**) *λ* = 1.15 mm, (**d**) *λ* = 1.225 mm, and (**e**) *λ* = 1.24 mm.

Figure 2a shows the transmission spectrum of the whistle-shaped MIM waveguide system with *s* = 30 nm, *w* = 50 nm, *d* = 10 nm, and *r*_2_ = 145 nm. As shown in Figure 2a, three asymmetrical profile peaks (0.645, 1.15, and 1.24 μm) can be observed in the transmission spectrum, which are regarded as the Fano resonance. The transmittance shows an unusual variation with the increase in wavelength from 1.15 to 1.24 μm; in particular, a steep slope curve can be observed in the transmission spectrum at the range of 1.225–1.24 μm. The *H*_z_ field distributions at *λ* = 0.645, 1.15, 1.225, and 1.24 mm were simulated and are displayed in Figure 2b–e to understand the physical mechanism of the asymmetrical profile peaks of the proposed structure. Steady stand wave modes can be observed, and the black arrows represent the time-average power flow distributions in the *H*_z_ field distribution graphs. For *λ* = 0.645 μm, Re(*n*_eff_) = 1.4435, and *λ*_spp_ = 0.4468 μm, so the number of wave nodes can be calculated by 2π*r*_2_/*λ*_spp_ = 2. As shown in Figure 2b, two wave nodes were formed in the *H*_z_ field distribution, and the time-average power flow distributions show an anticlockwise mode in the ring cavity. Most of the SPPs’ energy was limited in the whistle-shaped cavity, and part of the SPPs’ energy was coupled into the output waveguide, so a peak was formed in the transmission spectrum, as shown in Figure 2a. For *λ* = 1.24 μm, the Fano resonance was caused by the interference between the anticlockwise mode and the clockwise mode. The *H*_z_ field distribution (Figure 2e) is similar to Figure 2d (*λ*_2_ = 1.225 μm), but the SPPs’ energy could be coupled into the output waveguide and a resonance peak is formed in the transmission spectrum for *λ*_1_ = 1.24 μm. For *λ*_2_ = 1.225 μm, the SPPs’ energy was not coupled into the output waveguide. The time-average power flows show that an anticlockwise mode occurred. For *λ*_3_ = 1.15 μm, the *H*_z_ field distribution was mainly distributed in the whistle-shaped cavity, and part of SPPs was coupled into the output waveguide. The time-average power flows show a clockwise mode. Thus, the Fano resonance was due to the coupling between the anticlockwise and clockwise modes. For *λ*_4_ = 0.645 μm, the *H*_z_ field distribution reveals that a second-order vibration mode was formed in the whistle-shaped cavity, which was a new vibration mode. The time-average power flows show an anticlockwise mode.

**Figure 3:**
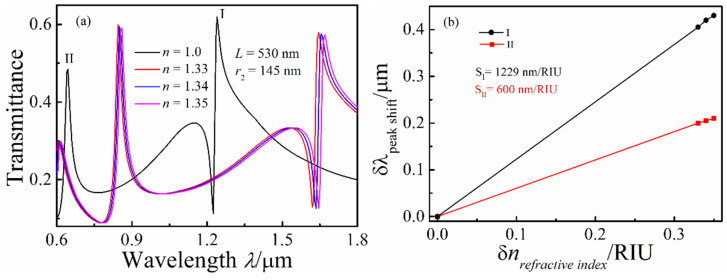
(**a**) Transmission spectra of the MIM waveguide-coupled whistle-shaped cavity with changing *n*; (**b**) shift of the Fano resonance peak as a function of the refractive index change *δn*.

The transmission spectra were simulated by replacing the air of the MIM waveguide system with different refractive index media (*n* = 1, 1.33, 1.34, and 1.35), which are shown in Figure 3a, to investigate the effect of refractive index *n* on the transmission properties of the whistle-shaped MIM waveguide system. As shown in Figure 3a, the Fano resonance peak red shifted with the increase in *n*, and another new resonance peak can be observed at the short wavelength range when *n* was larger than 1.33. The radius *r*_2_ of the whistle-shaped cavity was fixed because the *n*_eff_ value in the MIM waveguide-coupled whistle-shaped cavity decreased with the increase in *n*, and the number of modes increased. Therefore, a peak can be observed at the near short wave (0.60–0.7 μm) in the transmission spectra. We calculated the shift of the Fano resonance peaks (I and II) with the refractive index change. The sensitivity fitting curves of peaks I and II are shown in Figure 3b. The sensitivity of peak I is *S*_1_ = δ*λ*/δ*n* = 600 nm/RIU, and the sensitivity of peak II is *S*_2_ = δ*λ*/δ*n* = 1229 nm/RIU.

**Figure 4:**
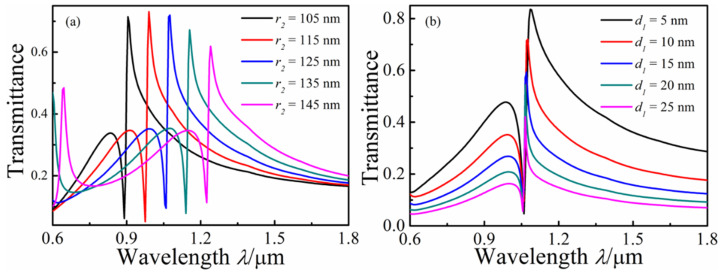
Transmission spectra of the MIM waveguide-coupled whistle-shaped cavity: (**a**) With changing *r*_2_; (**b**) with changing *d*_1_.

*r*_1_ was varied from 130 to 170 nm at intervals of 10 nm with *n* = 1, *w* = 50 nm, and *L* = 530 nm to study the effect of the different radii of the ring cavity on the Fano resonance of the MIM waveguide. With the increasing radius *r*_2_ of the ring cavity, red shifts of the transmission spectrum and decreases in the transmittances of the Fano resonance peak can be observed in Figure 4a. Figure 4b shows the transmission spectrum with different coupled distances *d* between the top waveguide and the whistle-shaped ring cavity, and the other parameters were fixed as *n* = 1, *r*_1_ = 150 nm, *r*_3_ = 100 nm, and *d*_1_ = 10 nm. From the transmission spectrum, the Fano resonance peak appears to be blue-shifted, but this was actually caused by the Fano resonance peak widening as *d*_1_ increased, and the transmittances decreased with the increase in *d*_1_. In contrast, the Fano resonance valley hardly shifted with as *d*_1_ increased, which was dependent on the length of the whistle-shaped ring cavity.

**Figure 5:**
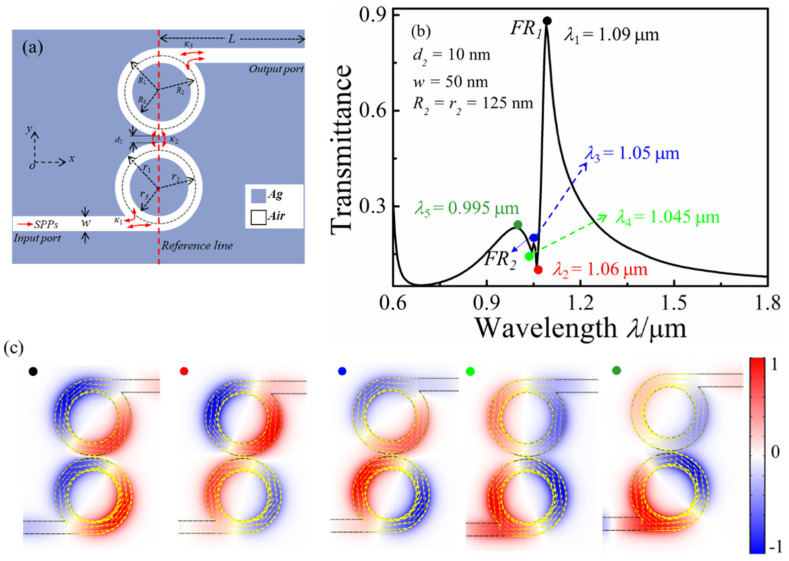
(**a**) Schematic of the MIM waveguide-coupled double whisper cavities; (**b**) transmission spectrum of the double whisper cavity structure; (**c**) *H*_z_ field distribution at different resonance wavelengths.

In this section, we investigate the derivative structure, MIM waveguide-coupled whistle-shaped cavity, and the proposed structure, as shown in Figure 5a. Figure 5b shows the transmission spectrum of the proposed MIM waveguide-coupled double whistle-shaped cavity, and the structural parameters were fixed at *n* = 1, *R*_2_ = *r*_2_ = 125 nm, and *d*_2_ = 10 nm. Although the transmission spectrum is similar to the MIM waveguide-coupled single-whisper cavity, another new peak emerged in the transmission spectrum. *FR*_2_ was unremarkable and *FR*_1_ and *FR*_2_ were extremely close when *R*_2_ = *r*_2_. The *H*_z_ field distributions at *λ*_1_ = 1.09 mm, *λ*_2_ = 1.06 mm, *λ*_3_ = 1.05 mm, *λ*_4_ = 1.045 mm, and *λ*_5_ = 0.995 mm are displayed in Figure 5c. For *λ*_1_ = 1.09 mm, the phase of the *H*_z_ field distribution in the bottom and top whistle-shaped cavities was opposite, and the output waveguide was located at the position of the strong negative time-averaged power flow, so the SPPs’ energy could be passed from the output waveguide. For *λ*_2_ = 1.06 mm, the Hz field distribution in the bottom and top whistle-shaped cavities was symmetric on the vertical axis. The output waveguide was located at the position joint between the positive and negative time-averaged power flows, so few SPPs’ energy could be passed. For *λ*_3_ = 1.05 mm, the strong *H*_z_ field distribution concentrated in the bottom whistle-shaped cavity, and few SPPs’ energy were coupled into the top whistle-shaped cavity. The output waveguide was located in the abdominal of the negative time-averaged power flow, so the SPPs’ energy could be passed to the output waveguide. For *λ*_4_ = 1.045 mm, the *H*_z_ field distribution in the bottom and top whistle-shaped cavities was located on the horizontal axis. For *λ*_5_ = 0.995 mm, the strong *H*_z_ field was mainly distributed in the bottom whistle-shaped cavity, and few SPPs’ energy were coupled into the top whistle-shaped cavity.

**Figure 6:**
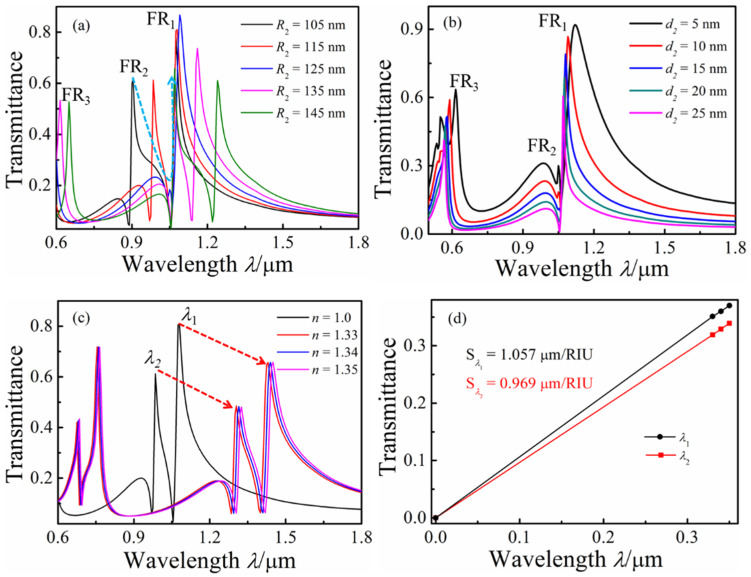
Transmission spectra of the different structural parameters at the MIM waveguide-coupled double whistle-shaped cavities: (**a**) *R*_2_ changing; (**b**) *d*_2_ changing; (**c**) *n* changing; (**d**) sensitivity.

The value of *R*_2_ changed and was varied from 130 to 170 nm at intervals of 10 nm, with *r*_1_ = 150 nm and *d*_2_ = 10 nm, to further investigate the effect of the top whisper cavity on the Fano resonance. A remarkable double Fano resonance was found in the transmission spectra, as shown in Figure 6a. With the increase in *R*_2_, an obvious red shift can be observed in the transmission spectra. The locations of the *FR*_2_ peak’s red shift with the increase in *R*_2_ and the transmittance of FR_2_ first showed decreases and then increases. When *R*_2_ = *r*_2,_ the transmittance was minimal, and with an increasing *R*_2_, new *FR*_3_ peaks can be observed in the transmission spectra for *R*_2_ = 135 nm. From Figure 6b, *FR*_1_ seems to be blue-shifted, but this was actually due to a decrease in the peak width of *FR*_1_ with the increase in the distance (*d*_2_) between the ring cavity from 5 to 25 nm when the values of *R*_2_ and *r*_2_ were fixed as 125 nm. The transmittance of *FR*_2_ decreased, but FR_2_ experienced no shift with as *d*_2_ increased. When *d*_2_ = 25 nm, the peak of *FR*_2_ disappeared in the transmission spectra. With an increasing *d*_2_, *FR*_3_ was blue-shifted and the transmittance decreased. Figure 6c shows the transmittance spectrum when changing its refractive index *n* (*n* = 1, 1.33, 1.34, and 1.35) of the proposed double whisper cavities, and the other parameters were fixed as *d*_2_ = 10 nm, *r*_2_ = 125 nm, and *R*_2_ = 115 nm. The Fano resonance exhibited a red shift with the increase in the refractive index *n,* and the sensitivities of 1.057 μm/RIU and 0.969 μm/RIU were obtained at *λ*_1_(*FR*_1_) and *λ*_2_(*FR*_2_).

## 4. Conclusions

In this work, a plasmonic refractive index sensor based on Fano resonance was proposed. The transmission properties were simulated numerically using FEM. Fano resonance was realized in the MIM waveguide-coupled whistle-shaped cavity. This phenomenon is due to the coupling of SPPs in the whistle-shaped cavity between the transmission along the clockwise and anticlockwise directions. Refractive index-sensing based on the Fano resonance was investigated by changing the refractive index of the insulator of the MIM waveguide. The results showed that a maximum sensitivity of 1229 RIU/nm was obtained. Compared to the single MIM waveguide-coupled whistle-shaped cavity, the double MIM with whistle-shaped cavity structure exhibited multi-Fano resonance. The refractive index sensor’s sensitivity was smaller than that of the single MIM waveguide-coupled whistle-shaped cavity. The results of this study will help in the design of new photonic devices and microsensors with high sensitivity.

## Data Availability

The data presented in this study are available upon request from the corresponding author. The data are not publicly available due to being supplied by Key Laboratory of Instrumentation Science & Dynamic Measurement (North University of China), Ministry of Education, and so cannot be made freely available.
